# Reliability of pedometer data in samples of youth and older women

**DOI:** 10.1186/1479-5868-4-4

**Published:** 2007-02-17

**Authors:** Lisa A Strycker, Susan C Duncan, Nigel R Chaumeton, Terry E Duncan, Deborah J Toobert

**Affiliations:** 1 Oregon Research Institute, 1715 Franklin Blvd, Eugene, OR 97403, USA

## Abstract

**Background:**

Pedometers offer researchers a convenient and inexpensive tool for objective measurement of physical activity. However, many unanswered questions remain about expected values for steps/day for different populations, sources of variation in the data, and reliability of pedometer measurements.

**Methods:**

This study documented and compared mean steps/day, demographic predictors of steps/day, and pedometer reliability in two longitudinal investigations, one involving a population-based youth sample (N = 367) and the other targeting postmenopausal women with type 2 diabetes (N = 270). Individuals were asked to wear pedometers (Yamax model SW-701) at the waist for 7 days and record steps/per day. They were also asked to record daily physical activities, duration, and perceived intensity (1 = low/light, 2 = medium/moderate, 3 = high/hard) for the same 7 days. In addition, survey data regarding usual physical activity was collected. Analyses of variance (ANOVA) were conducted to determine whether there were significant differences in pedometer results according to sex, age, and body mass index. Repeated measures ANOVAs were used to examine potential differences in results among differing numbers of days.

**Results:**

Mean steps/day were 10,365 steps in the youth sample and 4,352 steps in the sample of older women. Girls took significantly fewer steps than boys, older women took fewer steps than younger women, and both youth and women with greater body mass took fewer steps than those with lower body mass. Reliability coefficients of .80 or greater were obtained with 5 or more days of data collection in the youth sample and 2 or more days in the sample of older women. Youth and older women were more active on weekdays than on weekends. Low but significant associations were found between step counts and self-report measures of physical activity in both samples.

**Conclusion:**

Mean steps/day and reliability estimates in the two samples were generally consistent with previously published studies of pedometer use. Based on these two studies, unsealed pedometers were found to offer an easy-to-use and cost-effective objective measure of physical activity in both youth and older adult populations.

## Background

Among objective monitors of physical activity available to researchers, pedometers are perhaps the most convenient and inexpensive [1]. However, the science of pedometer monitoring is in its infancy [2]. Many unanswered questions remain about expected values for steps/day for different populations, sources of variation in the data, and reliability of pedometer measurements.

### Expected steps/Day

A number of studies of physical activity in children have yielded mean daily step counts ranging from about 7,000 to 15,000, depending on the child's age and sex, the brand of pedometer used, and the study design [3-7]. Vincent et al. [8] found mean step counts ranging from15,673–18,346 for Swedish boys, 13,864–15,023 for Australian boys, 12,554–13,872 for American boys, 12,041–14,825 for Swedish girls, 11,221–12,322 for Australian girls, and 10,661–11,383 for American girls. Few studies have established steps/day norms in adult populations and in special populations (e.g., the chronically ill). In N = 493 Swiss adults, Sequeira et al. [9] found a decline in activity from about 12,000 to about 7,000 in men and from about 9,000 to about 7,000 in women between ages 25–34 and 65–74 years. In Japanese adults, Hatano [10] found that steps declined from about 8,200 to about 7,200 from ages 30–39. Tudor-Locke et al. [2] reported 10,082 steps/day in adults (mean age = 38). Mean steps/day have been shown to be lower in diseased and disabled populations, about 5,000 steps/day for patients with peripheral arterial occlusive disease [11] and patients with maleolar fractures [12], and about 6,000 steps/day for individuals with type 2 diabetes [13].

### Sources of variation in pedometer data

Research has linked pedometer data with participant characteristics, such as sex [3,7,6,8,4], age [7,4], and body mass [8,2,14]. It was hypothesized in the present investigation that youth would take more steps/day than older women (and younger-age youth/women would take more steps/day than older-age youth/women), that boys would take more steps/day than girls, and that body mass would be negatively associated with steps/day.

### Reliability

Most published studies of pedometer reliability have found that at least 3 days of data collection are necessary for adequate reliability (α = 0.80 or greater), but additional days increases reliability [15,3,2]. Trost et al. [15] concluded that 4–5 days of measurement, using accelerometers, were necessary to assess usual activity in children. Rowe et al. [3] found that 6 days of pedometer data were reliable for researching habitual physical activity. Reliability appears to be improved by including weekdays and weekends in the monitoring period [3,2] and by adjusting for seasonal effects [2]. The present study hypothesized that 4–5 days of pedometer data with youth and 3 days with older women would be necessary to attain reasonable reliability.

### Relations between pedometer data and self-report measures of activity

A few studies have attempted to link objective pedometer data with more subjective measures of physical activity, with mixed results. Cardon and De Bourdeaudhuij [6] found a moderate correlation between step counts and reported minutes of activity on self-report diaries. Wilde et al. [7] found that pedometer measurements were related to reported levels of activity. Rowe et al. [3] found no relation between pedometers and an exercise questionnaire in children. Research comparing pedometers with indirect calorimetry has found that the pedometer underestimated energy expenditure [16] and suggested that pedometers provide accurate measurements for walking speeds from 3–4 mph but are less accurate at slower speeds. In the present study, small but significant associations between pedometer data and self-reported physical activity were hypothesized.

The purpose of the current investigation is to add to the existing literature on pedometer assessment by documenting mean steps/day and pedometer reliability in two samples: a population-based youth sample and a sample of postmenopausal women with type 2 diabetes.

## Methods

### Participants

#### Youth sample (N = 367)

Data were collected from 10-, 12-, and 14-year-old youth randomly recruited in a metropolitan area in the Pacific Northwest and [17,18]. Of eligible families, 68% agreed to participate. Participants were assessed annually for 4 years. Data from T1 were used for the present analyses.

#### Adult sample (N = 270)

Data were collected from postmenopausal women with type 2 diabetes who received their medical care from participating primary care clinics [19-21]. All patients meeting basic eligibility criteria were sent letters signed by their primary care providers describing the study and recommending participation. Of those women meeting eligibility criteria, 51% agreed to participate and completed the initial assessment. Participants were assessed four times over 2 years (i.e., baseline, 6 months, 12 months, 24 months). The intervention components in this study are detailed elsewhere [20]. The present investigation examined baseline assessment data only.

Appropriate Institutional Review Board approval for research with human subjects was obtained for both samples.

### Procedures

In the youth sample, target children completed in-home surveys under the supervision of research assistants to ensure privacy. In the adult sample, women completed surveys at a central assessment site. In both samples, trained assessors measured height (m) and weight (kg) of participants using calibrated, sensitive scales.

#### Seven-day physical activity record

A 7-day physical activity record was developed specifically for these two studies based on our past experience. For 7 days, target children and women were asked to complete a daily record of physical activities. The form was structured so that each day participants could separately record the type of activities in which they engaged (e.g., walking, jogging, aerobic activity, swimming), the perceived intensity of each activity (1 = low/light, 2 = medium/moderate, 3 = high/hard), and the number of minutes engaged in each activity. Based on these reports, a physical activity summary variable was created by multiplying frequency by duration by intensity of activities. The use of diaries to collect activity data has well-documented limitations, including possible misinterpretation of questions, difficulty for participants in recalling the time or intensity of activities, or deliberate misrepresentation (over-reporting), but self-report techniques are low-cost, have low participant burden, and are an acceptable method of assessing physical activity behavior as long as the limitations are recognized and/or in concert with more objective measures [22].

#### Pedometer

At the assessment visit, target children and women were shown how to wear a pedometer and record the number of steps taken each day for 7 days. Children, who were usually assessed in the evening, were instructed to start wearing the pedometer the following day; women, who were usually assessed in the morning, were instructed to start wearing the pedometer immediately. In both studies, participants were instructed to clip the pedometer to the waistline above the right knee each morning, to wear the pedometer all day while doing usual activities, and to remove the pedometer and record the day's steps at night before resetting the device and going to bed. All pedometers were unsealed, as in Rowe et al. [3]. The Yamax Digiwalker SW-701 (Yamax Corporation, Japan) was chosen for these studies because an identical model with fewer features (SW-200) performed best in a research study when compared to other pedometers [23]. From the pedometer data, the first and last days were excluded because of partial data collection and an average steps/day variable was computed by summing the number of steps for up to 5 days and dividing by the number of days for which pedometer information was recorded (if 5 days of data were not provided, this construct was still calculated based on fewer days).

#### Physical activity survey items

In the youth sample, children were asked how many times per week and for how many months over the past year they were involved in a list of 26 activities (including bicycling, running, soccer, and walking) both in school and outside school. This activity list was created especially for the study to create summary constructs of total school-related and nonschool-related activity. In addition, based on previously validated measures [24,25], including the Youth Risk Behavior Surveillance System (YRBSS), youth were asked: "On how many of the past 7 days did you exercise or take part in hard physical activities that made you sweat and breathe hard for at least 20 minutes without stopping?", "On how many of the past 7 days did you exercise or take part in moderate physical activities that increased your breathing a bit for a total of at least 30 minutes during the day?", and "In a typical week, how many days do you take part in any regular physical activity long enough to work up a sweat (heart beats rapidly)?". For these three items, responses ranged from 0 to 7 days. A fourth item asked, "Compared to others of your same age and sex, how much physical activity do you get?" Individuals responded on a 5-point scale ranging from (1) Much less than others to (5) Much more than others.

In the adult sample, the previously validated Community Healthy Activities Model for Seniors (CHAMPS) Activities Questionnaire for Older Adults [26] was used to measure physical activity. The CHAMPS, a widely used measure shown to be sensitive to change in similar populations, is a 45-item, self-report instrument that assesses frequency per week over the past 6 months of specific activities in the areas of social, recreation and hobbies, work around the house, walking and jogging, swimming, stretching exercises, and other types of exercise. Respondents indicate the number of times per week the activity is performed and the total time on average spent doing the activity each week. Based on these responses, constructs were created summarizing frequency of moderate physical activity and frequency of all activity (including sedentary activity).

### Analyses

Descriptive statistics (i.e., means, standard deviations, frequencies, skewness, and kurtosis) were computed for all variables to pinpoint outliers, understand the nature of the data, and ensure that distributions met assumptions of the statistical tests being used. Pedometer results were split by gender (in the youth sample), age (in both samples), and four levels of body mass index (in both samples). Age was split according to the three cohorts in the youth sample (10, 12, and 14 years) and in four decade ranges in the adult sample (40–49, 50–59, 60–69, and 70–79 years). Body mass index was split into four quartiles for analyses, with each quartile representing roughly a fourth of each sample. Reliability (Cronbach's α) coefficients were computed separately for 2, 3, 4, and 5 days of pedometer data. Analyses of variance (ANOVA) were conducted to determine whether there were significant differences in pedometer results according to sex, age, and body mass index. Repeated measures ANOVAs were used to examine potential differences in results among differing numbers of days. T-tests were used to compare mean weekday vs. weekend pedometer counts. Pearson product-moment correlation coefficients were computed to examine the strength of relations between pedometer counts and self-report measures of physical activity. As with all measures used in this study, distributions of the self-report measures were examined for skewness and kurtosis prior to analysis to ensure that their distributions were approximately normal and appropriate for the statistical tests used.

## Results

### Sample characteristics

Mean body mass index, calculated as weight (kg) divided by height (m) squared, was 22.5 (SD = 5.6) kg/m^2 ^in the youth sample and 35.4 (SD = 8.3) kg/m^2 ^in the sample of older women. For analyses, this variable was split into four quartiles in the samples of youth (lower than 18 kg/m^2^, 18–20 kg/m^2^, 20–25 kg/m^2^, more than 25 kg/m^2^) and older women (29.9 kg/m^2 ^or lower, 30–39 kg/m^2^, 40–49 kg/m^2^, 50 or more kg/m^2^).

In the sample of older women, 24% had only a high school or lower education while 66% reported having at least some college. About half of the sample (54%) reported an annual income of less than $30,000 with the rest of the sample (46%) reporting an income of $30,000 or greater. Education and income data were not available in the youth sample.

### Self-reported physical activity

In self-reported physical activity, youth averaged a score of 260 (SD = 204) on the summary variable reflecting frequency (times/week) multiplied by duration (minutes) multiplied by intensity of physical activities on their 7-day diaries. From survey items, youth reported an average 327 (SD = 332) bouts of physical activity in school over the past year, 450 (SD = 532) bouts of nonschool physical activity over the past year, 3.5 (SD = 1.9) days per week of hard physical activity, 4.2 (SD = 2.0) days per week of moderate physical activity, 4.0 (SD = 1.9) days of regular physical activity in a typical week, and a rating of 3.5 on a 5-point scale reflecting "about the same" or "somewhat more" physical activity than others. These statistics suggest a moderately active youth sample.

Based on a 7-day physical activity diary, older women averaged a score of 14.6 (SD = 20.7) on the variable reflecting minutes of activity multiplied by intensity over the past week, indicating a low level of activity. The women averaged 5.0 (SD = 6.4) bouts of moderate activity per week and 16.7 (11.4) bouts of all activity per week, including sedentary activities, based on their CHAMPS survey responses estimating activity over the past 6 months.

### Steps/Day

Of 367 youth participants, 362 provided at least 1 day of pedometer data (N = 308 gave 5 full days, N = 31 provided 4 days, N = 19 provided 3 days, N = 2 gave 2 days, and N = 2 recorded 1 day). All of the women in the older adult sample provided at least 2 days of pedometer data (N = 212 provided all 5 days, N = 45 recorded 4 days, N = 11 provided 3 days, and N = 2 gave 2 days). In both samples, all participants (except the 2 youth with only 1 day of data) reported pedometer totals on at least 1 weekend day, most commonly Saturday, and 1 weekday. Mean steps/day measured by pedometers for the two samples are presented in Table 1. As shown, youth took 10,365 steps/day (SD = 4,178) compared to 4,352 steps/day (SD = 2,981) in the sample of chronically ill older women.

**Table 1:** Mean Values of Baseline Steps/Day for Youth and Older Women

	Mean Steps/Day (SD)	*p *Value
Youth Sample (N = 367)		
All	10,365 (4,178)	
Gender		< .001
Male (N = 183)	11,283 (4,257)	
Female (N = 184)	9,472 (3,909)	
Age		.43
10-year-olds (N = 116)	9,916 (3,406)	
12-year-olds (N = 124)	10,465 (4,266)	
14-year-olds (N = 127)	10,562 (4,528)	
Body Mass Index (kg/m^2^)		.02
18.00 and lower (N = 70)	11,005 (3,796)	
18.01 to 20.00 (N = 64)	10,621 (4,354)	
20.01 to 25.00 (N = 142)	10,641 (4,507)	
25.01 and higher (N = 88)	9,190 (3,546)	
Older Women Sample (N = 270)		
All	4,352 (2,981)	
Age		.01
40- to 49-year-olds (N = 18)	4,359 (2,300)	
50- to 59-year-olds (N = 101)	5,047 (3,271)	
60- to 69-year-olds (N = 98)	3,888 (2,572)	
70 years and older (N = 53)	3,773 (3,051)	
Body Mass Index (kg/m^2^)		< .001
29.9 and lower (N = 78)	5,860 (3,309)	
30 to 39.9 (N = 123)	4,263 (2,894)	
40 to 49.9 (N = 54)	2,930 (1,653)	
50 and higher (N = 13)	2,148 (1,402)	

### Sources of variation in pedometer data

Analyses of variance results indicated a significant effect for gender in the youth sample (*F*(1,365) = 18.02, *p *< .001) with girls taking fewer steps than boys.

In the youth sample, age was not a significant predictor of mean steps/day. Since Trost et al. [15] found differences in the variability of pedometer data in children, with less variability in adolescents; the Levene Test for Homogeneity of Variance was conducted in the present study to examine potential difference in pedometer variance estimates. Ten-year-olds were found to have significantly (*p *< .05) lower variance in the steps/day variable than 12- and 14-year-olds.

In the sample of older women, age was important (*F*(3,281) = 3.62, *p *= .014) in that women younger than 60 years of age recorded significantly more steps per day on their pedometers than older women.

In both samples, body mass index was strongly associated with pedometer results (youth sample: *F*(3,360) = 3.22, *p *= .023. sample of older women: *F*(3,280) = 15.93, *p *< .001). Steps/day were lower for those in the groups with greatest body mass.

### Reliability

Reliability results for pedometer data are presented in Table 2. Reliability coefficients ranged from .73 (2 days) to .82 (5 days) in the youth sample, and from .84 (2 days) to .87 (5 days) in the sample of older women.

**Table 2:** Reliability Coefficients and Inter-Day Comparisons of Pedometer Data for Youth and Older Women

	Youth Sample		Older Women Sample	
	Value	*p*	Value	*p*
Reliability Coefficients				
2 Days	.73		.84	
3 Days	.78		.86	
4 Days	.78		.85	
5 Days	.82		.87	
Inter-Day Differences				
By Day		.84		.98
Day 1 Mean Steps	10,182		4,446	
Day 2 Mean Steps	10,380		4,477	
Day 3 Mean Steps	10,465		4,420	
Day 4 Mean Steps	10,127		4,558	
Day 5 Mean Steps	10,425		4,466	
By Weekday/Weekend		<.001		.01
Weekday Mean Steps	10,599		4,742	
Weekend Mean Steps	9,373		4,291	

Analyses of variance showed no significant differences in steps/day among the 5 days of data collection in either sample.

In both samples, weekdays were significantly more active than weekends.

### Relations between pedometer data and self-report measures of activity

As shown in Table 3, moderate but significant relations were found between step counts and self-report measures of activity (*r *= .15 to .36), with the exception of school physical activity in youth.

**Table 3:** Relations Between Pedometer Data and Self-Report Measures of Activity for Youth and Older Women

	Youth Sample *r (p)*	Older Women Sample *r (p)*
7-Day Step Average with:		
7-Day Self-Reported Activity (Diary)	.28 (<.001)	.31 (<.001)
School-related Physical Activity (Survey)	.04 (.49)	
Nonschool Physical Activity (Survey)	.15 (.003)	
Frequency Hard Exercise (Survey)	.25 (<.001)	
Frequency Moderate Exercise (Survey)	.21 (<.001)	.36 (<.001)
Frequency All Activity (Survey)		.32 (<.001)
Usual Physical Activity (Survey)	.19 (<.001)	
Activity Compared to Others (Survey)	.20 (<.001)	

## Discussion

Pedometers are easy to use and relatively inexpensive, which makes them attractive measurement tools for large-scale studies. More elaborate activity monitors, such as accelerometers, may cost 20 times as much and require special software. But questions remain about pedometer measurement, including norms for different populations, sources of variation, and instrument reliability. The present investigation sought to address these questions in a population-based youth sample and a sample of chronically ill older women. This is one of very few studies reporting pedometer results in which randomized sampling techniques were used for recruitment.

Mean steps/day in the two samples were generally consistent with previously published studies of pedometer use, and were consistent with our hypothesis that youth would take more steps/day than older women.

Youth in this study recorded about 10,000 steps/day, as similarly reported among high school students in Wilde et al. [7] and among elementary school children in Vincent and Pangrazi [4]. Considering that boys with 13,000 steps and girls with 12,000 steps engage in about 60 minutes or more of moderate activity [27] – which is recommended for healthy youth most days of the week – it is clear that young people in the current study as a group did not meet the recommendation. Only 28% of the boys and girls in the present investigation reached the recommended level of daily activity.

Chronically ill older women in this study recorded fewer than 5,000 steps/day, which is similar to other findings among diseased older women (e.g., [13,11]). Translating steps into activity levels for adults, Tudor-Locke and Bassett [28] provide the following indices: <5000 steps/day = "sedentary lifestyle," 5000–7499 steps/day = "low active," 7500–9999 = "somewhat active," 10000–11,499 steps/day = "active," and >12,500 steps/day = "highly active." Based on these categorizations, the women in the current study on average fell into the sedentary range, well short of the 30 minutes of moderate activity recommended most days of the week. Only 15% of the women reached the somewhat active level.

Although pedometer totals were self-reported in the samples of youth and older women, the consistency of results with previously published studies, as well as anecdotal reports from field researchers, suggests that users generally wore the devices as directed and reported steps accurately. Criterion-referenced pedometer data is necessary for public health policymakers to establish practical step-count standards for people of all ages and abilities, and researchers should continue to investigate steps/day norms among youth and older women in both general and special populations.

As hypothesized, and as reported in several previous youth studies (e.g., [8,4]), girls in this study accumulated fewer steps/day than boys. Also, as hypothesized and previously reported (e.g., [14]), greater body mass index was associated here with fewer steps/day among older women and youth. Increasing age was found to be related to fewer steps in the sample of older women, but not in the youth sample, possibly because of the restricted range. Curiously, unlike Trost et al. [15], significantly lower variances in step counts were found in the current study for 10-year-olds compared to 12- and 14-year-olds; further research is needed to better understand age-based variability of step data among youth. Relations between steps and sociodemographic characteristics have practical implications for researchers and practitioners designing prevention and intervention programs. The demographic and physiological variables used in this study were limited, however; future research should continue to investigate these and other possible sources of variation in pedometer data.

In terms of reliability, the present study found that at least 5 days of pedometer data were needed in the youth sample (4–5 days were hypothesized), consistent with previously published reports, including Trost et al. [15], who suggested 4–5 days of measurement, to assess usual activity in children using objective measures. This study found that just 2 days of pedometer data were needed in the sample of older women (compared to our hypothesis of 3 days) to obtain α coefficients of at least .80, which is a relatively small participant burden compared to a week of monitoring. Little reliability appears to be gained by additional data collection, but there are other advantages to longer sampling periods (e.g., to gain an estimate of year-round habitual activity and seasonal effects, as in Tudor-Locke et al., [2]).

Reactivity is always a concern with any measurement tool, and especially among biofeedback devices like pedometers which are known to motivate change. The monitoring process itself could cause participants to take more steps/day, since the pedometer focuses attention on activity, provides feedback, and may be affected by a socially desirable response set. None of the pedometers in the samples of youth or older women were sealed. Participants were told that the pedometers would count their steps and that this would indicate their activity. This study found that steps/day totals were consistent across the 5-day period. While steps remained consistent across the 5 days of monitoring, the monitoring process may have caused participants to take more steps/day every day. True reactivity can not be accurately assessed without a pre-monitoring measurement for comparison, which is a limitation of this study. Well-designed studies of pedometer reactivity should be pursued, using random and representative samples, sample sizes with sufficient power to detect effects, and methods (e.g., interrupted time series) to compare pedometer step counts with activity levels measured prior to pedometer use. The latter point is especially important, as this provides the only means to determine the extent to which participants react, or change behavior, once they start wearing a pedometer. The present study included monitoring on weekends and weekdays to accurately reflect all types of activity.

Regarding criterion validity, correlations between pedometer totals and self-reported physical activity were low but significant, as hypothesized, suggesting that the two modes of measurement provide both common and unique information. Interestingly, one simple survey item in this study (i.e., days of hard physical activity per week) associated almost as strongly as the 7-day physical activity diary with pedometer totals in the youth sample. The implication is that a young person's physical activity may be estimated nearly as well by asking one clear question as by demanding a full week of diary-keeping. This is a promising finding that merits further study.

It may be that pedometer reliability and validity are affected by sample characteristics. The present study was not powered to test differences in reliability and validity within subgroups; however, exploratory analyses were conducted to identify trends for more study. Reliability of pedometer counts tended to differ by age (i.e., better reliability at older ages) but not body mass in the youth sample, and was fairly consistent across age and weight groups in the adult sample. Relations between pedometer counts and self-reported activity as recorded on the 7-day diary tended to differ by age and body mass in both samples (i.e., strongest relations at younger ages and lower body mass in the youth sample; strongest correlations at older ages and mid-level body mass in the adult sample). Finally, correlations between pedometer counts and physical activity survey variables tended to vary in the youth and adult samples by weight (i.e., strongest relations in lowest-weight individuals); relations did not differ by age in the youth sample but tended to be stronger in the older-age women. We encourage researchers to further analyze pedometer reliability and construct validity in subgroups to better understand the possible effects of gender, age, body mass, and other variables.

Despite the consistency of step counts across these two samples with previously published research, anecdotal reports indicate that the pedometers performed poorly for specific individuals. Field researchers reported that the pedometers seemed to work best when recording steps during moderate to vigorous walking or running, but failed to count movement among overweight and/or disabled participants (especially the older women) who moved slowly, or with activities lacking vigorous lower-body movement (e.g., pilates, yoga, slow bicycling). Yamax pedometers have been found to underestimate energy expenditures at speeds of 2 mph or less [29]. The pedometers also undercounted activity for participants engaged in exercises during which the devices could not be worn (e.g., swimming, a popular activity among older women). In both populations, pedometers were lost when they popped off during activity or under stress at the waistline. Even so, compliance in wearing the pedometer was remarkably good, with most women and youth providing at least 4 full days of step counts (85% in the youth sample and 95% in the sample of older women).

Based on our experience across these two studies, we find unsealed pedometers to be an easy-to-use and inexpensive objective measure of physical activity in samples of both youth and older women, warranting continued use in research and practice. These studies showed that 2 days of recording pedometer data for older women and 5 days for youth provide highly reliable estimates when worn across weekdays and weekends. Despite some loss of pedometers, which were replaced if necessary, most participants complied in wearing the device for at least 4 days. Step totals recorded by the pedometers in the two populations were as expected, and in the expected direction. Correlations between the pedometer results and physical activity self-reports were low, but significant. Future research is needed to investigate reactivity of pedometers more fully, examine differences in variance in step counts especially in youth, and study prospective predictors of step counts in different populations.
